# Supplementary material for: The genetic and phenotypic correlates of mtDNA copy number in a multi-ancestry cohort
Source: HGG Adv. 2023 May 9;4(3):100202. doi: 10.1016/j.xhgg.2023.100202 (PMC10225932; doi:10.1016/j.xhgg.2023.100202)
Supplement: Document S1. Figures S1–S11 [file mmc1.pdf]

HGGA, Volume *4*

Supplemental information

The genetic and phenotypic correlates

# of mtDNA copy number in a multi-ancestry cohort

Arslan A. Zaidi, Anurag Verma, Colleen Morse, Penn Medicine BioBank, Marylyn D. Ritchie, and Iain Mathieson

References

1. Rath, S., Sharma, R., *et al.* MitoCarta3.0: an updated mitochondrial proteome now with sub-organelle localization and pathway annotations. *Nucleic Acids Research* **49,** D1541–D1547 (D1 2021).
2. Taylor, R. W. & Turnbull, D. M. Mitochondrial DNA mutations in human disease. *Nature Reviews Genetics* **6,** 389–402 (5 2005).
3. Chinnery, P. F. Primary Mitochondrial Disorders Overview. *GeneReviews®* (2021).
4. D'Erchia, A. M., Atlante, A., *et al.* Tissue-specific mtDNA abundance from exome data and its correlation with mitochondrial transcription, mass and respiratory activity. *Mitochondrion* **20,** 13–21 (2015).
5. Bentlage, H. A. & Attardi, G. Relationship of Genotype to Phenotype in Fibroblast-derived Trans-mitochondrial Cell Lines Carrying the 3243 Mutation Associated with the Melas Encephalomyopathy: Shift towards Mutant Genotype and Role of mtDNA Copy Number. *Human Molecular Genetics* **5,** 197–205 (2 1996).
6. Lee, S. R., Heo, H. J., *et al.* Low abundance of mitochondrial DNA changes mitochondrial status and renders cells resistant to serum starvation and sodium nitroprusside insult. *Cell Biology International* **39,** 865–872 (7 2015).
7. Rausser, S., Trumpff, C., *et al.* Mitochondrial phenotypes in purified human immune cell subtypes and cell mixtures. *eLife* **10** (2021).
8. Picard, M. Blood mitochondrial DNA copy number: What are we counting? *Mitochondrion* **60,** 1–11 (2021).
9. Filograna, R., Mennuni, M., *et al.* Mitochondrial DNA copy number in human disease: the more the better? *FEBS Letters* **595,** 976–1002 (8 2021).
10. Hägg, S., Jylhävä, J., *et al.* Deciphering the genetic and epidemiological landscape of mitochondrial DNA abundance. *Human Genetics* **140,** 849–861 (2021).
11. Longchamps, R. J., Yang, S. Y., *et al.* Genome-wide analysis of mitochondrial DNA copy number reveals loci implicated in nucleotide metabolism, platelet activation, and megakaryocyte proliferation. *Human Genetics* **141,** 127–146 (2022).
12. Knez, J., Winckelmans, E., *et al.* Correlates of Peripheral Blood Mitochondrial DNA Content in a General Population. *American Journal of Epidemiology* **183,** 138–146 (2015).
13. Howes, R. E., Patil, A. P., *et al.* The global distribution of the Duffy blood group. *Nature Communications 2011 2:1* **2,** 1–10 (1 2011).
14. Reich, D., Nalls, M. A., *et al.* Reduced Neutrophil Count in People of African Descent Is Due To a Regulatory Variant in the Duffy Antigen Receptor for Chemokines Gene. *PLoS Genetics* **5** (ed Visscher, P. M.) e1000360 (2009).
15. Ng, Y. S. & Turnbull, D. M. Mitochondrial disease: genetics and management. *Journal of Neurology* **263,** 179–191 (1 2016).

16. Parikh, S., Goldstein, A., *et al.* Diagnosis and management of mitochondrial disease: A consensus statement from the Mitochondrial Medicine Society. *Genetics in Medicine* **17,** 689–701 (9 2015).

17. Suomalainen, A. & Battersby, B. J. Mitochondrial diseases: The contribution of organelle stress responses to pathology. *Nature Reviews Molecular Cell Biology* **19,** 77–92 (2 2018).

18. Russell, O. M., Gorman, G. S., *et al.* Mitochondrial Diseases: Hope for the Future. *Cell* **181,** 168–188 (1 2020).

19. Agarwal, S., Fulgoni, V. L., *et al.* Assessing alcohol intake & its dosedependent effects on liver enzymes by 24-h recall and questionnaire using NHANES 2001-2010 data. *Nutrition Journal* **15,** 1–12 (1 2016).

20. Mansouri, A., Gaou, I., *et al.* An alcoholic binge causes massive degradation of hepatic mitochondrial DNA in mice. *Gastroenterology* **117,** 181–190 (1 1999).

21. Zhang, Y., Guallar, E., *et al.* Association between mitochondrial DNA copy number and sudden cardiac death: Findings from the Atherosclerosis Risk in Communities study (ARIC). *European Heart Journal* **38,** 3443–3448 (2017).

22. Wiersma, M., van Marion, D. M., *et al.* Cell-Free Circulating Mitochondrial DNA: A Potential Blood-Based Marker for Atrial Fibrillation. *Cells* **9** (5 2020).

23. Ashar, F. N., Zhang, Y., *et al.* Association of mitochondrial DNA copy number with cardiovascular disease. *JAMA Cardiology* **2,** 1247–1255 (2017).

24. El-Hattab, A. W. & Scaglia, F. Mitochondrial DNA Depletion Syndromes: Review and Updates of Genetic Basis, Manifestations, and Therapeutic Options. *Neurotherapeutics* **10,** 186–198 (2 2013).

25. Yang, J., Lee, S. H., *et al.* GCTA: a tool for genome-wide complex trait analysis. *American journal of human genetics* **88,** 76–82 (1 2011).

26. Genovese, G., Friedman, D. J., *et al.* Association of trypanolytic ApoL1 variants with kidney disease in African Americans. *Science* **329,** 841–5 (2010).

27. Limou, S., Nelson, G. W., *et al.* APOL1 kidney risk alleles: population genetics and disease associations. *Adv Chronic Kidney Dis* **21,** 426–33 (2014).

28. Chen, M. H., Raffield, L. M., *et al.* Trans-ethnic and Ancestry-Specific Blood-Cell Genetics in 746,667 Individuals from 5 Global Populations. *Cell* **182,** 1198–1213.e14 (5 2020).

29. Cai, N., Li, Y., *et al.* Genetic Control over mtDNA and Its Relationship to Major Depressive Disorder. *Current Biology* **25,** 3170–3177 (24 2015).

30. Chong, M., Mohammadi-Shemirani, P., *et al.* GWAS and ExWAS of blood Mitochondrial DNA copy number identifies 71 loci and highlights a potential causal role in dementia. *eLife* **11** (2022).

31. Zaidi, A. A. & Makova, K. D. Investigating mitonuclear interactions in human admixed populations. *Nature Ecology and Evolution* **3,** 213–222 (2019).

32. Crawford, N., Prendergast, D., *et al.* Divergent Patterns of Mitochondrial and Nuclear Ancestry Are Associated with the Risk for Preterm Birth. *The Journal of Pediatrics* **194,** 40–46.e4 (2018).

33. Udler, M. S., Nadkarni, G. N., *et al.* Effect of genetic African ancestry on EGFR and kidney disease. *Journal of the American Society of Nephrology* **26,** 1682–1692 (2015).

34. Zilbermint, M., Hannah-Shmouni, F., *et al.* Genetics of Hypertension in African Americans and Others of African Descent. *International Journal of Molecular Sciences* **20,** 1081 (2019).

35. Samokhvalov, A. V., Irving, H., *et al.* Alcohol consumption, unprovoked seizures, and epilepsy: A systematic review and meta-analysis. *Epilepsia* **51,** 1177–1184 (7 2010).

36. Wu, L. T., Woody, G. E., *et al.* Racial/Ethnic Variations in Substance-Related Disorders Among Adolescents in the United States. *Archives of General Psychiatry* **68,** 1176–1185 (11 2011).

37. Holmbeck, M. A., Donner, J. R., *et al.* A Drosophila model for mito-nuclear diseases generated by an incompatible interaction between tRNA and tRNA synthetase. *DMM Disease Models and Mechanisms* **8,** 843–854 (8 2015).

38. Burton, R. S., Ellison, C. K., *et al.* The sorry state of F2 hybrids: consequences of rapid mitochondrial DNA evolution in allopatric populations. *The American naturalist* **168,** S14–S24 (2006).

39. Meiklejohn, C. D., Holmbeck, M. A., *et al.* An Incompatibility between a Mitochondrial tRNA and Its Nuclear-Encoded tRNA Synthetase Compromises Development and Fitness in Drosophila. *PLoS Genetics* **9** (ed Payseur, B. A.) e1003238 (1 2013).

40. Reinhardt, K., Dowling, D. K., *et al.* Medicine. Mitochondrial replacement, evolution, and the clinic. *Science* **341,** 1345–6 (2013).

41. Chou, J.-Y. & Leu, J.-Y. The Red Queen in mitochondria: cyto-nuclear co-evolution, hybrid breakdown and human disease. *Frontiers in Genetics* **6,** 187 (2015).

42. Morrow, E. H., Reinhardt, K., *et al.* Risks inherent to mitochondrial replacement. *EMBO reports* **16,** 541–544 (5 2015).

43. Claiborne, A. B., English, R. A., *et al.* Finding an ethical path forward for mitochondrial replacement. *Science* **351,** 668–670 (6274 2016).

44. Eyre-Walker, A. Mitochondrial Replacement Therapy: Are Mito-nuclear Interactions Likely To Be a Problem? *Genetics* **205,** 1365–1372 (4 2017).

45. Trumpff, C., Michelson, J., *et al.* Stress and circulating cell-free mitochondrial DNA: A systematic review of human studies, physiological considerations, and technical recommendations. *Mitochondrion* **59** (2021).

46. Denny, J. C., Ritchie, M. D., *et al.* PheWAS: demonstrating the feasibility of a phenome-wide scan to discover gene–disease associations. *Bioinformatics* **26,** 1205–1210 (9 2010).

47. Bastarache, L. Using Phecodes for Research with the Electronic Health Record: From PheWAS to PheRS. *Annual review of biomedical data science* **4,** 1–19 (2021).

48. Venables, W. N. & Ripley, B. D. *Modern Applied Statistics with S* Fourth. ISBN 0-387-95457-0 (Springer, New York, 2002).

49. R Core Team. *R: A Language and Environment for Statistical Computing* R Foundation for Statistical Computing (Vienna, Austria, 2021).

50. Li, H. A statistical framework for SNP calling, mutation discovery, association mapping and population genetical parameter estimation from sequencing data. *Bioinformatics* **27,** 2987 (21 2011).

51. García-Olivares, V., Muñoz-Barrera, A., *et al.* A benchmarking of human mitochondrial DNA haplogroup classifiers from whole-genome and whole-exome sequence data. *Scientific Reports* **11,** 1–11 (2021).

52. Weissensteiner, H., Pacher, D., *et al.* HaploGrep 2: mitochondrial haplogroup classification in the era of high-throughput sequencing. *Nucleic Acids Research* **44,** W58–W63 (W1 2016).

53. Fast two-stage phasing of large-scale sequence data. *American Journal of Human Genetics* **108,** 1880–1890 (2021).

54. Maples, B. K., Gravel, S., *et al.* RFMix: a discriminative modeling approach for rapid and robust local-ancestry inference. *American journal of human genetics* **93,** 278–88 (2013).

55. Auton, A., Abecasis, G. R., *et al.* A global reference for human genetic variation. *Nature 2015 526:7571* **526,** 68–74 (7571 2015).

56. Alexander, D. H., Novembre, J., *et al.* Fast model-based estimation of ancestry in unrelated individuals. *Genome research* **19,** 1655–64 (2009).

57. Das, S., Forer, L., *et al.* Next-generation genotype imputation service and methods. *Nature Genetics* **48,** 1284–1287 (10 2016).

58. Chang, C. C., Chow, C. C., *et al.* Second-generation PLINK: rising to the challenge of larger and richer datasets. *GigaScience* **4,** s13742–015–0047–8 (1 2015).

59. Shriner, D., Adeyemo, A., *et al.* Joint Ancestry and Association Testing in Admixed Individuals. *PLOS Computational Biology* **7,** e1002325 (12 2011).

60. Plummer, M., Best, N., *et al.* CODA: Convergence Diagnosis and Output Analysis for MCMC. *R News* **6,** 7–11 (2006).

Supplementary material

Penn Medicine Biobank Team and Contributions

Leadership

Daniel J. Rader, M.D., Marylyn D. Ritchie, Ph.D., Michael D. Feldman M.D.
Contribution: Contributed to securing funding, study design and oversight.

Patient Recruitment and Regulatory Oversight

JoEllen Weaver, Nawar Naseer, Ph.D., M.P.H., Afiya Poindexter, Ashlei Brock, Khadijah Hu-Sain, Yi-An Ko
Contributions: JW manages patient recruitment and regulatory oversight of study. NN manages participant engagement, assists with regulatory oversight, and researcher access. AP, AB, KH, YK perform recruitment and enrollment of study participants.

Lab Operations

JoEllen Weaver, Meghan Livingstone, Fred Vadivieso, Ashley Kloter, Stephanie DerOhannessian, Teo Tran, Linda Morrel, Ned Haubein, Joseph Dunn
Contribution: JW, ML, FV, SD conduct oversight of lab operations. ML, FV, AK, SD, TT, LM perform sample processing. NH, JD are responsible for sample tracking and the laboratory information management system.

Clinical Informatics

Anurag Verma, Ph.D., Colleen Morse, P.T, D.P.T, M.S, Marjorie Risman, M.S., Renae Judy, B.S.
Contribution: All authors contributed to the development and validation of clinical phenotypes used to identify study subjects and (when applicable) controls.

Genome Informatics

Anurag Verma Ph.D., Shefali S. Verma, Ph.D., Yuki Bradford, M.S., Scott Dudek, M.S., Theodore Drivas, M.D., PH.D.
Contribution: A.V., S.S.V. are responsible for the analysis, design, and infrastructure needed to quality control genotype and exome data. Y.B. performs the analysis. T.D. and A.V. provides variant and gene annotations and their functional interpretation of variants.

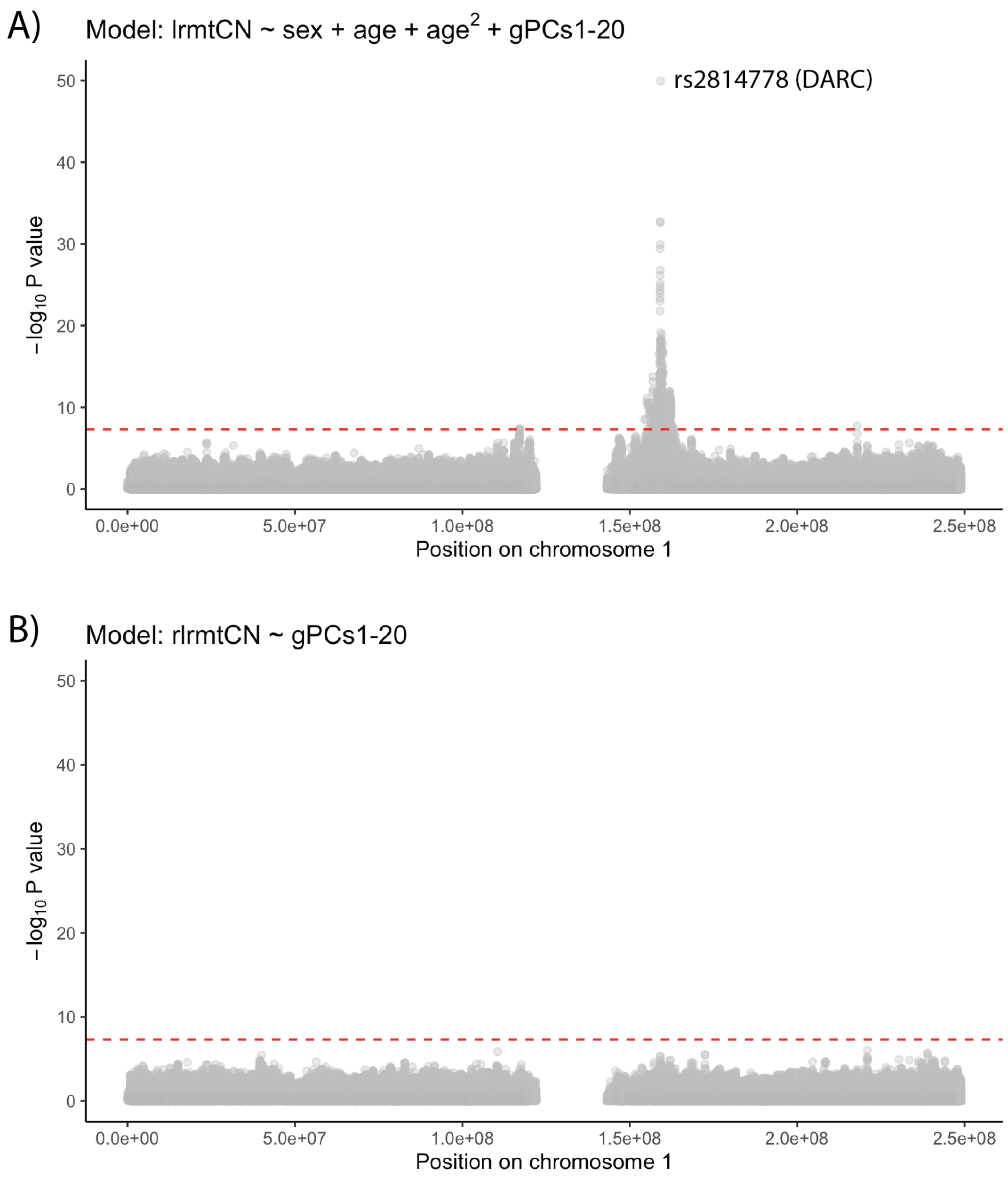

Figure S1: Manhattan plot of GWAS on rmtCN (A) before and (B) after correction for blood cell counts in the AFR cohort. Only chromosome 1 is displayed.

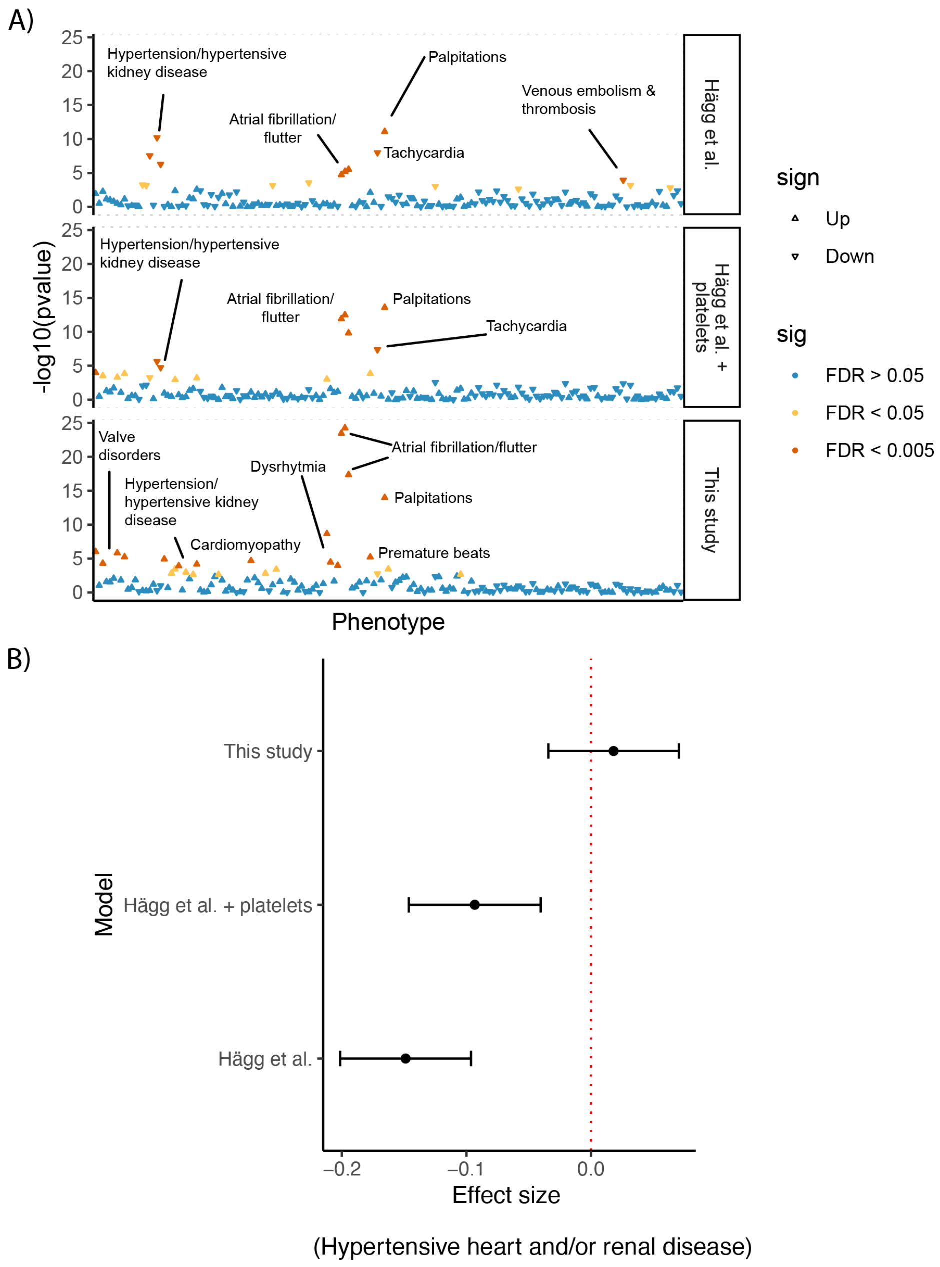

Figure S2: Sensitivity of association of mtDNA copy number with cardiac phenotypes to model choice. (A) The x-axis shows phenotypes arranged in order of phecode number such that similar phenotypes cluster together, and the y-axis shows the negative log of the association p-value. (Top panel) A model closely mimicking that used by Hägg *et al.* where, in addition to lrmtCN, we include sex, age, age^2, neutrophil %, lymphocyte %, total white blood cell count, and 20 PCs as predictors. (Middel panel) The same as previous model except for the addition of platelet count as a covariate. (Bottom panel) The model used in this study where we use rlrmtCN (residuals from the model described in the main text), sex, age, and age^2, and 20 PCs as predictors. (B) Forest plot illustrating the change in effect size for one phenotype (hypertension and renal disease) in the EUR cohort.

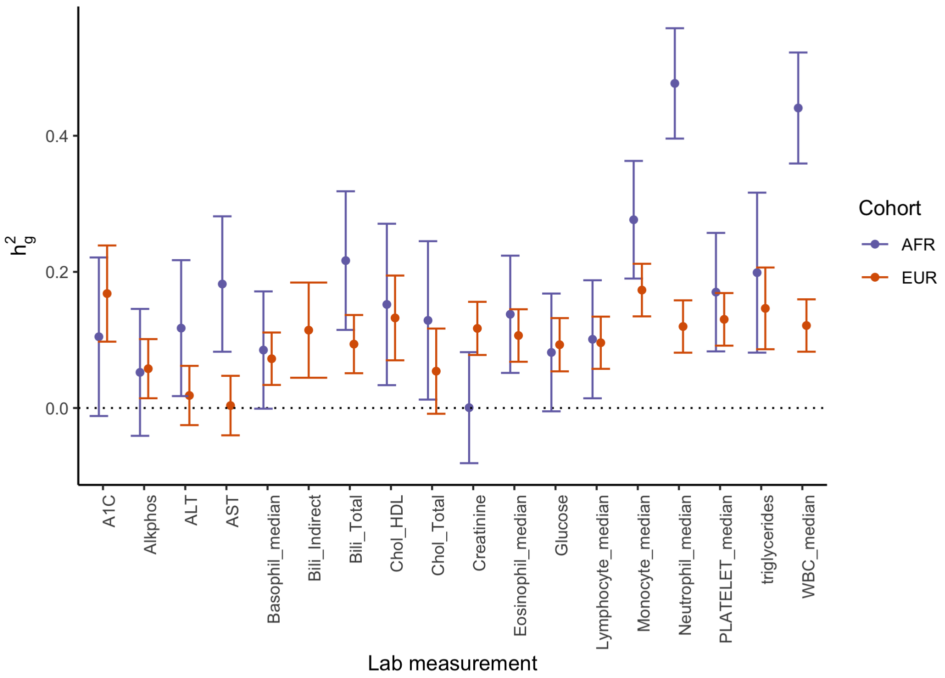

Figure S3: The heritability of lab measurements in the PMBB shown separately for the AFR and EUR cohort. Only lab measurements where the lower bound of the 95% CI was greater than 0 in at least in one of the cohorts is shown

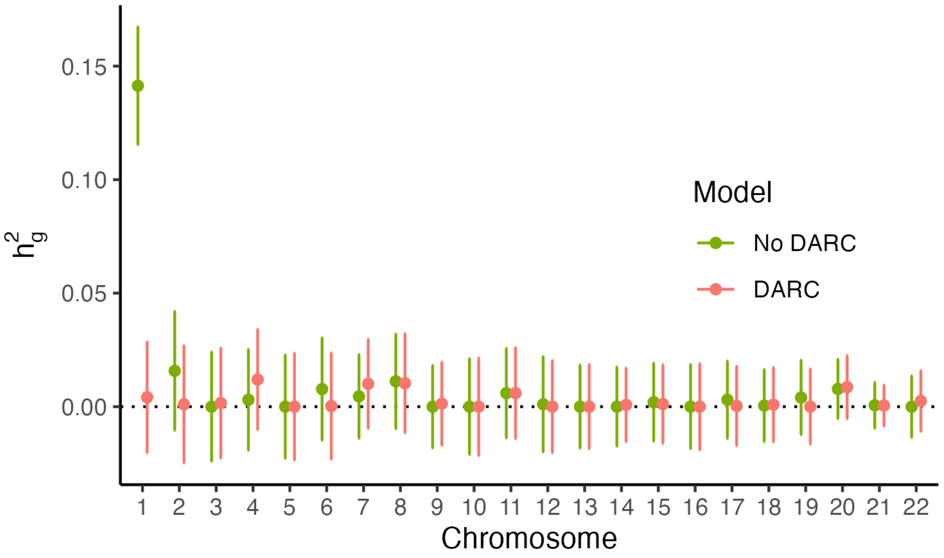

Figure S4: Heritability of neutrophil count partitioned by chromosome in the AFR cohort. The colors represent two models with and without genotype at the Duffy-null allele as a covariate. Both models included sex, age, age^2, and 20 PCs as covariates.

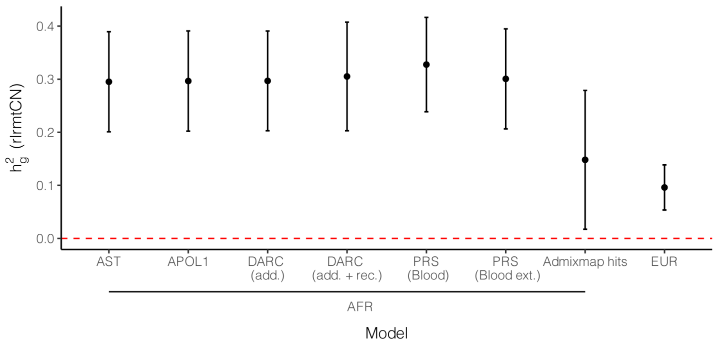

Figure S5: SNP heritability of rlrmtCN in the AFR (first 7 columns) and EUR (last columns) cohorts estimated using GCTA. All models included 20 genetic PCs calculated separately in each cohort. For the AFR cohort, the heritability was estimated with additional covariates: AST = amino aspartate transferase levels; APOL1 = genotype at the APOL1 locus; DARC (add.) = genotype at the rs2814778 SNP coded additively; DARC (add. + rec.) = additive and recessive coding for rs2814778; PRS (Blood) = polygenic risk scores for blood counts which were measured in the PMBB; PRS (Blood ext.) polygenic risk scores for an extended set of blood traits (see Methods for details).

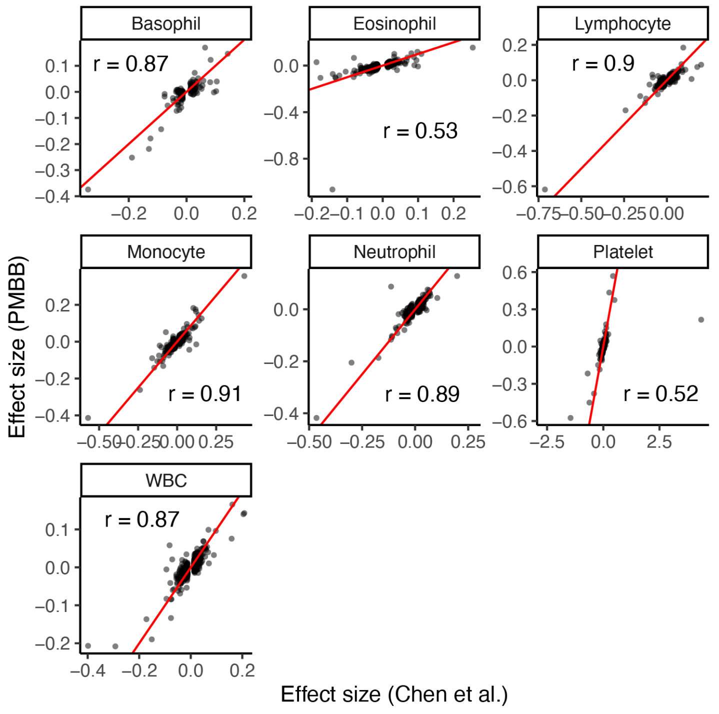

Figure S6: Effect sizes for variants discovered in Chen *et al.* [28] are correlated with their effects estimated in the PMBB EUR cohort. The red line represents $y = x$. The effects could only be re-estimated for traits which were available in the PMBB. One variant which can be seen as having a large effect size on platelet counts as estimated by Chen *et al.* was removed (see Methods for more details). The numbers in each plot show the correlation coefficients.

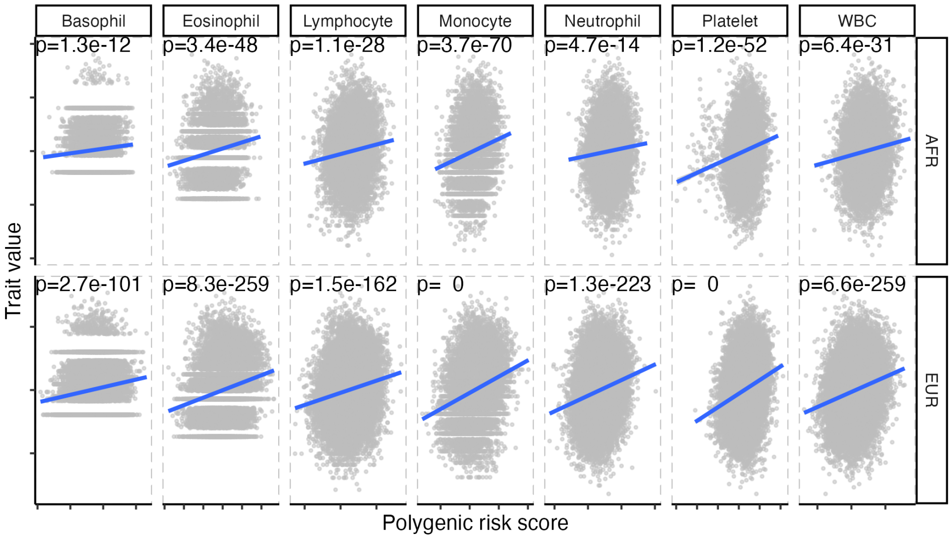

Figure S7: Polygenic risk scores (PRS) constructed using effects from Chen *et al.* [28] are strongly correlated with the actual phenotypes in both PMBB cohorts. The blue line represents the linear regression line.

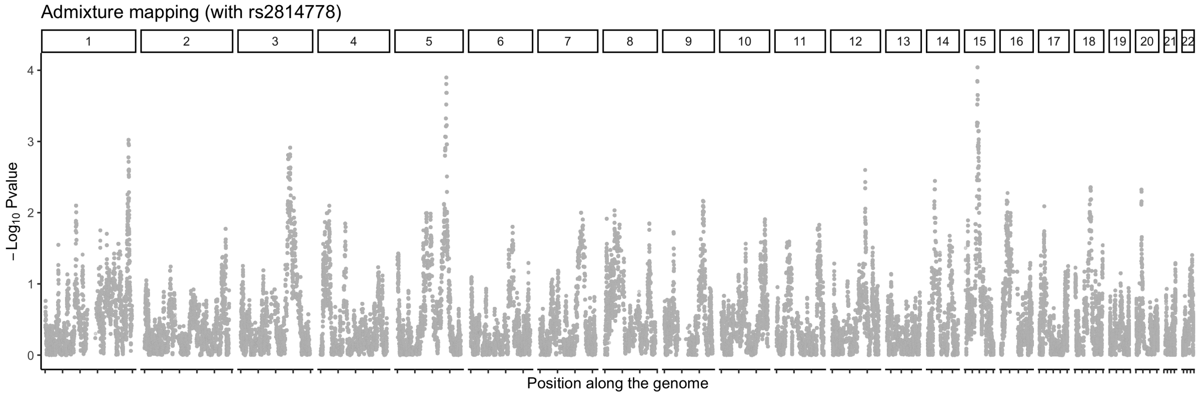

Figure S8: Admixture mapping of rlrmtCN in the AFR cohort. The x-axis shows the position along the genome and the y-axis shows the -$\log_{10}$ of the p-value of association between local ancestry at each position and rlrmtCN. Global ancestry proportion and the Duffy-null genotype were included as covariates.

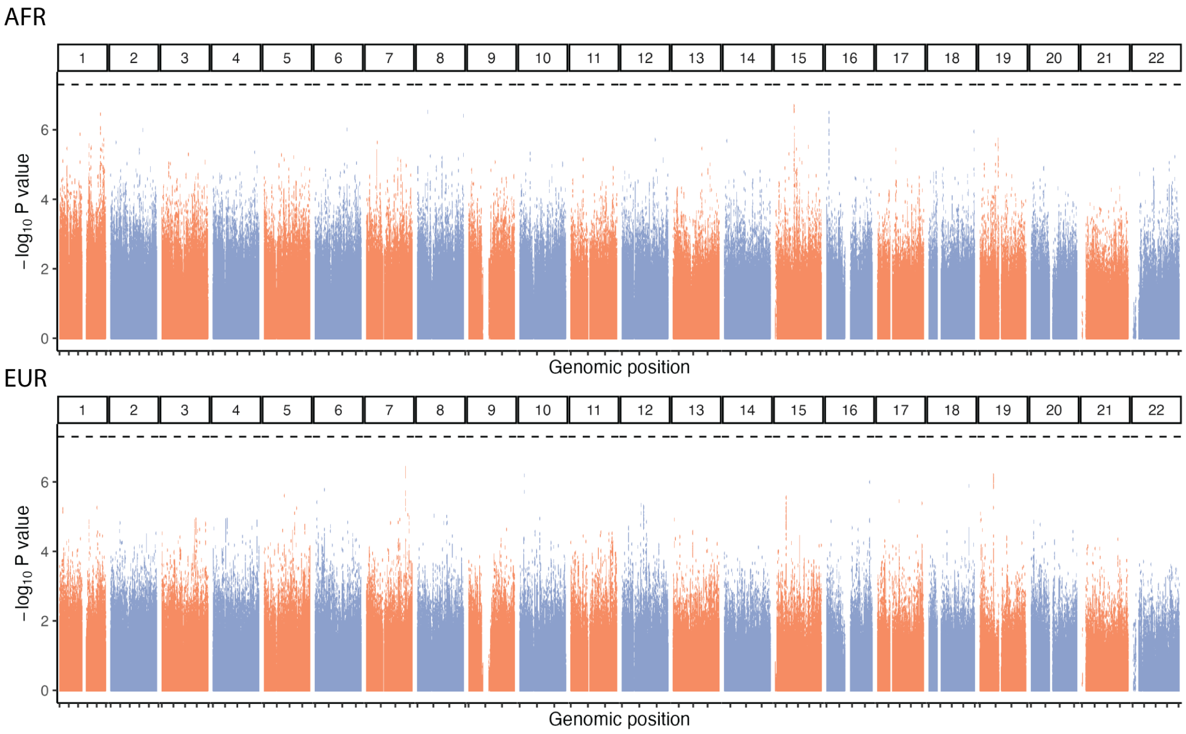

Figure S9: GWAS of mtDNA copy number (rlrmtCN) carried out separately in the AFR and EUR cohorts. The x-axis shows the genomic position, grouped by chromosomes (vertical panels) and the y-axis shows the -$\log_{10}$ of the association p-value. The dotted horizontal line represents the genome-wide significance threshold of 5×10^{-08}. The first 20 PCs, computed separately within each cohort, were included as covariates.

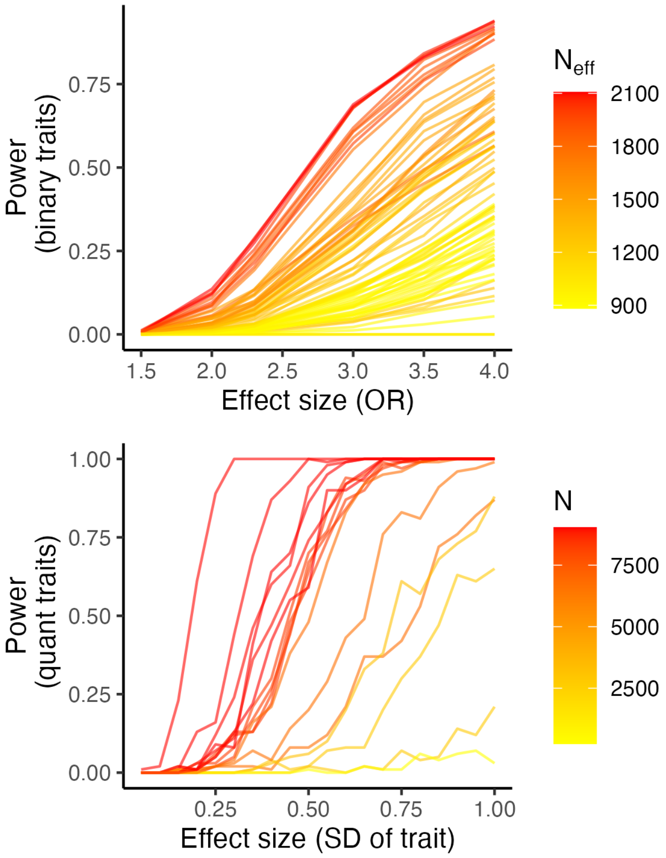

Figure S10: Power to detect a significant interaction effect between mitochondrial and nuclear ancestry for binary traits (case/control data) and quantitative traits (e.g. lab measurements). The x-axis lists the effect size, i.e., odds ratio (OR) or in units of standard deviation, for binary and quantitative traits, respectively and the y-axis shows the power of detecting an interaction effect at $\alpha = 5 \times 10^{-05}$. For quantitative traits, the color represents the sample size and for binary traits, it represents the effective sample size (N_{eff}): $n\phi(1-\phi)$ where ϕ is the proportion of cases and n is the sample size.

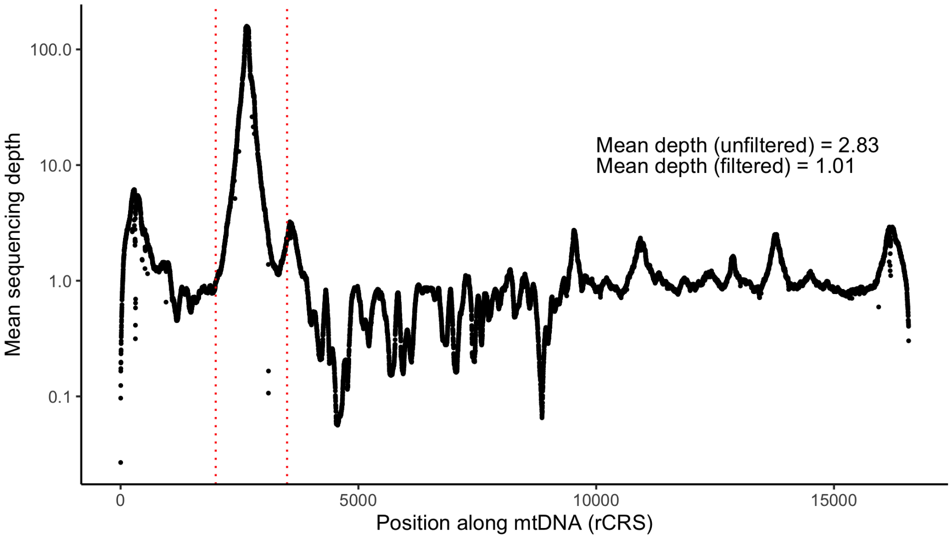

Figure S11: Mean sequencing depth (across individuals in the PMBB) of off-target reads aligning to the Revised Cambridge Reference Sequence (rCRS) of human mtDNA. Note that the y-axis is on a log-scale. Depth values from the region between the dotted red lines were filtered out for subsequent analysis.
